# Special Issue “Genetics and Epigenetics in Endocrine Disorders”

**DOI:** 10.3390/genes14091763

**Published:** 2023-09-05

**Authors:** Katarina Trebušak Podkrajšek, Primož Kotnik

**Affiliations:** 1 Laboratory for Translational Medical Biochemistry, Institute of Biochemistry and Molecular Genetics, Faculty of Medicine, University of Ljubljana, Vrazov trg 2, 1000 Ljubljana, Slovenia; 2 Clinical Institute for Special Laboratory Diagnostics, University Children’s Hospital, University Medical Centre Ljubljana, Vrazov trg 1, 1000 Ljubljana, Slovenia; 3 Department of Endocrinology, Diabetes, and Metabolic Diseases, University Children’s Hospital, University Medical Centre Ljubljana, Bohoriceva Ulica 20, 1000 Ljubljana, Slovenia; primoz.kotnik@mf.uni-lj.si; 4 Chair of Pediatrics, Faculty of Medicine, University of Ljubljana, Vrazov trg 2, 1000 Ljubljana, Slovenia

In the last decade, the development of high-throughput sequencing methodologies has significantly improved the gathering of genomic information and consequent under-standing of the genetic and epigenetic background of complex and monogenetic endocrine disorders. This knowledge importantly enables the identification of the etiological factors involved in the pathogenic mechanism in endocrine disorders and is becoming essential for clinicians for the diagnostic and consequent personalized management of individual patients. Nevertheless, understanding of the detailed molecular pathology underlying endocrine disorders is still incomplete and requires further elucidation.

This Special Issue reviews the current knowledge of some of the important pathophysiological mechanisms underlying the etiology of type 1 diabetes. It highlights the interplay between genetic predisposition and other non-genetic factors, such as viral infections, diet, and gut biome [1]. This Issue opens up certain paths to some innovative perspectives possibly important for the future treatment of type 1 diabetes. This Special Issue also describes some novel insights into genetic variability in long non-coding RNA associated with diabetic retinopathy [2] and possible islet regeneration through treatment with garlic extract in diabetic rats [3].

Translating scientific discoveries into better management or new treatment options is an enormous challenge, and most rare diseases do not yet have approved treatment. Nevertheless, state-of-the-art analytical tools in genomics, proteomics, and metabolomics have advanced this quest significantly. The first step is to clarify the genetic etiology of individual disorders. In this Special Issue, we elucidate the genetic etiology in combined pituitary hormone deficiency in consanguineous Sudanese families [4], in idiopathic short stature [5], in Klinefelter syndrome [6], and in NNT-related primary adrenal insufficiency [7], which greatly facilitates the search for etiological elucidation of these rare disorders. However, genetic background has a major impact on endocrine manifestations not only in monogenic disorders but also in complex diseases, as illustrated in morbid obesity [8].

On the other hand, epigenetic factors are thought to play an important role in complex disorders, and therefore their role in type 1 diabetes [9] and osteoporosis [10] is reviewed in detail. Moreover, the presented interactome of PTH-regulated miRNAs and their predicted target genes may facilitate further understanding of the mechanism of action of PTH on bone metabolism and the development of new therapeutic targets.

Of course, some major obstacles to obtaining a deep understanding of the genetic and epigenetic etiology of endocrine disorders remain unaddressed. These include the interpretation of large structural variants and noncoding variants that often remain un-interpreted because of the current lack of knowledge and tools in the field. In addition, the clinical variability of patients is importantly challenging the interpretation of the results of genetic and epigenetic studies.
